# Minisatellite instability is found in colorectal tumours with mismatch repair deficiency

**DOI:** 10.1054/bjoc.2001.2058

**Published:** 2001-11

**Authors:** M G Coleman, A C Gough, D J Bunyan, D Braham, D M Eccles, J N Primrose

**Affiliations:** University Department of Surgery, Department of Clinical Genetics, Southampton General Hospital, Tremona Road, Southampton, SO16 6YD, UK; Wessex Regional Genetics Laboratory, Salisbury District Hospital, Salisbury, wiltshire, SP2 8BJ, UK

**Keywords:** colorectal cancer, hereditary non-polyposis dolorectal cancer, microsatellite instability, minisatellite, MVR-PCR

## Abstract

Microsatellite instability (MSI) in colorectal tumours is demonstrated by PCR amplification of several different microsatellite loci. Minisatellites, which are repeats of longer sequences also found throughout the genome, may also be affected by tumorigenesis. Certain minisatellite alleles contain 2 types of similar repeat unit that are randomly interspersed. The interspersion pattern can be analysed by mapping variant repeat units along an amplified allele, minisatellite variant repeat unit mapping PCR (MVR-PCR). We have applied microsatellite analysis with 10 markers and MVR-PCR for locus D7S21 to 33 cases of colorectal neoplasia, 27 sporadic and 6 from patients suspected of having hereditary non-polyposis colorectal cancer (HNPCC). Of the 27 sporadic cases, 3 were MSI-high on microsatellite analysis and one MSI-low. Instability with MVR-PCR was observed, but only in the MSI-high cases. Four of the HNPCC patients had mismatch repair (MMR) gene mutations in either hMLH1 or hMSH2. All 4 had DNA instability by MVR-PCR, but only two of these had MSI (one high, one low). The other 2 of the 6 patients with suspected HNPCC were negative to mutation analysis. One had features strongly suggestive of HNPCC and was unstable by both microsatellite analysis (MSI-high) and by MVR-PCR. The other tumour, from an Amsterdam criteria positive kindred, did not demonstrate instability by any technique. Thus MVR-PCR detects DNA instability in MSI-high sporadic tumours and in those associated with HNPCC where MSI is observed. Further, in some MMR mutation positive cases MSI was not seen but instability was observed by MVR-PCR. MVR-PCR may be a valuable adjunct to the detection of MMR deficiency in colorectal tumours and it may allow new insights into the nature of DNA instability in this condition.© 2001 Cancer Research Campaign   http://www.bjcancer.com


*British Journal of Cancer* (2001) **85**(10), 1486–1491
© 2001 Cancer Research Campaign
doi: 10.1054/ bjoc.2001.2058, available online at http://www.idealibrary.com on IDEAL

### Minisatellite instability is found in colorectal tumours with mismatch repair deficiency

**MG Coleman[1], AC Gough[1], DJ Bunyan[3], D Braham[1], DM Eccles[2] and JN Primrose[1]**

[1]University Department of Surgery and [2]Department of Clinical Genetics, Southampton General Hospital, Tremona Road, Southampton, SO16 6YD, UK;
[3]Wessex Regional Genetics Laboratory, Salisbury District Hospital, Salisbury, Wiltshire, SP2 8BJ, UK

**Summary** Microsatellite instability (MSI) in colorectal tumours is demonstrated by PCR amplification of several different microsatellite loci. Minisatellites, which are repeats of longer sequences also found throughout the genome, may also be affected by tumorigenesis. Certain minisatellite alleles contain 2 types of similar repeat unit that are randomly interspersed. The interspersion pattern can be analysed by mapping variant repeat units along an amplified allele, minisatellite variant repeat unit mapping PCR (MVR-PCR). We have applied microsatellite analysis with 10 markers and MVR-PCR for locus D7S21 to 33 cases of colorectal neoplasia, 27 sporadic and 6 from patients suspected of having hereditary non-polyposis colorectal cancer (HNPCC). Of the 27 sporadic cases, 3 were MSI-high on microsatellite analysis and one MSI-low. Instability with MVR-PCR was observed, but only in the MSI-high cases. Four of the HNPCC patients had mismatch repair (MMR) gene mutations in either hMLH1 or hMSH2. All 4 had DNA instability by MVR-PCR, but only two of these had MSI (one high, one low). The other 2 of the 6 patients with suspected HNPCC were negative to mutation analysis. One had features strongly suggestive of HNPCC and was unstable by both microsatellite analysis (MSI-high) and by MVR-PCR. The other tumour, from an Amsterdam criteria positive kindred, did not demonstrate instability by any technique. Thus MVR-PCR detects DNA instability in MSI-high sporadic tumours and in those associated with HNPCC where MSI is observed. Further, in some MMR mutation positive cases MSI was not seen but instability was observed by MVR-PCR. MVR-PCR may be a valuable adjunct to the detection of MMR deficiency in colorectal tumours and it may allow new insights into the nature of DNA instability in this condition. © 2001 Cancer Research Campaign http://www.bjcancer.com

**Keywords:** colorectal cancer; hereditary non-polyposis dolorectal cancer; microsatellite instability; minisatellite; MVR-PCR

The adenoma to carcinoma sequence (Fearon and Vogelstein, 1990) may, in some cases, be accelerated by DNA instability in cancer cells which are acquired as a result of mutations in DNA mismatch repair genes (Fishel et al, 1993; Papodopoulos et al, 1994) or their epigenetic silencing (Kane et al, 1997). The DNA mismatch repair (MMR) gene products recognise and repair mismatched DNA sequences. The loss of normal DNA repair mechanisms in a cell results in numerous widespread additions, deletions and substitutions during cell division. This change, known as DNA instability was originally identified in tumours from patients with colorectal cancer (Ionov et al, 1993). It has been found subsequently to be associated with hereditary non-polyposis colorectal cancer (HNPCC) (Aaltonen et al, 1994) and has since been also identified in some sporadic tumours (Thibodeau et al, 1993). DNA instability is conventionally detected by the PCR amplification of repetitive microsatellite sequences found scattered throughout the genome. Length mutation in these oligonucleotide repeats is termed microsatellite instability (MSI) (Thibodeau et al, 1993). Until recently, no uniform consensus existed for the detection of microsatellite instability (MSI) (Boland et al, 1998) and since its discovery in 1993, a profusion of approaches for MSI detection have evolved leading to difficulty in comparing results (Bocker et al, 1997). By 1997, this resulted in the rationalisation of the method for MSI detection (Dietmaier et al, 1997) and the recommendation by an international consensus panel of a group of microsatellite loci to be examined (Boland et al, 1998). With time, the utility of the recent international consensus in dealing with these difficulties will become apparent.

Minisatellites are also repetitive DNA sequences, but the repeat length is 9–45 base pairs in length. Minisatellite alleles display remarkable variability in the repeat unit sequence that may be analysed using PCR (minisatellite variant repeat unit mapping, MVR-PCR) (Nakamura et al, 1987). Somatic mutation of minisatellite allele length has been observed in tumour cell lines prior to the discovery of MSI or its association with MMR genes (Armour et al, 1989). However, there has been no systematic examination of minisatellite mutation in colorectal cancer nor is it known whether any such mutations are related to MSI.

The hypervariable D7S21 locus has 405 bp of flanking DNA and contains two minisatellite alleles (MS31A and MS31B) separated by 15 bp (Neil and Jeffreys, 1993). MS31A repeat units have two adjacent sites of base substitutional polymorphism; G or A followed by C or T, and all repeat units are 20 bp long. These features satisfy the criteria for MVR-PCR.

MVR-PCR reactions consist of two separate reactions each containing a repeat unit specific primer (31-TAG II-AC or GT), a primer specific to a fixed point in the MVR flanking DNA (31A), and a driver primer (TAG II) specific to an extension on the repeat unit specific primer. At low concentrations, the repeat unit specific primers 31-TAG II-AC or GT attach to one repeat unit subtype per target minisatellite molecule and extend into the flanking DNA. Priming also occurs from the flanking DNA creating a sequence with an end complementary to the TAG II primer. Amplification

*Received 27 March 2001*
*Revised 21 June 2001*
*Accepted 11 July 2001*

*Correspondence to:* JN Primrose now occurs efficiently with high concentrations of TAG II and the 31A to create a stable set of products extending from the flanking DNA to each repeat unit subtype (AC or GT).

The aim of this work was to determine whether minisatellite instability was manifest in colorectal cancer, specifically in patients known to have MSI. In this paper we provide evidence that DNA instability may be detected by MVR-PCR including in cases with a MMR gene mutation where MSI has not been observed.

### METHODS

### Patients

Patients undergoing resection with sporadic colorectal carcinoma were selected consecutively. Fresh samples of tumour and normal colonic mucosa were taken at the time of surgery and snap frozen in liquid nitrogen. Patients with known HNPCC also had fresh samples taken at the time of resection from index cases with colorectal cancer. Clinical details and histology were recorded.

### DNA extraction

Genomic DNA was extracted by a variation of the phenol/chloroform method. Briefly, sections were ground and suspended in 200 μl phosphate-buffered saline (PBS), proteinase K (200 μg $ml^{-1}$, ICN, Basingstoke, UK) and 0.5% N lauryl sarcosine (BDH, Poole, UK). They were incubated for 3 hours at 55˚C with 300 μl water saturated phenol (Lancaster Synthesis, Morecambe, UK), extracted twice, and chloroform (BDH) extracted and precipitated with twice the volume of 100% ethanol and 0.03 M sodium acetate (pH 5.3). The pellet was washed with 1 ml of 70% ethanol, air dried and resuspended in 100 μl × 1 Tris-ethylene diamine tetra-acetic acid (Tris-EDTA) pH8.0. DNA quality was checked on a 0.6% agarose gel by electrophoresis. Quality was confirmed by control PCR for a fragment of the glyceraldehyde-phosphate dehydrogenase (GAPDH) gene: 0.2 mM dNTPs (deoxynucleotide triphosphates) (Pharmacia Biotech, St Albans, UK), 1 × PCR buffer (Biogene Ltd, Cambridge UK), 1.5 mM $MgCl_2$, 2% DMSO (dimethyl sulfoxide) (Sigma, Poole, UK), 75 ng of forward and reverse primer specific for the GAPDH gene (5′-AGTACGCT-GCAGGGCCTCACTCCTT-3′, 3′-AAGAGCCAGTCTCTGGC-CCCAGCCA-5′, Cruachem, Glasgow, UK), 0.5 units Bio/Polythermase™ (Biogene) and 0.5 μl genomic DNA. Sterile water was added to each mix in a 0.5 ml microfuge tube to make up a final volume of 25 μl. Tubes were placed in an automatic thermal cycler (Techne, Cambridge, UK) and denatured for 3 minutes at 94˚C followed by 35 repeated cycles of 94˚C for 30 seconds, annealing at 58˚C for 30 seconds and extension at 72˚C for 30 seconds. This was followed by a final elongation step at 72˚C for 2 minutes. A negative PCR reaction control (using sterile water in place of genomic DNA) was used in all reactions. 10 μl of each product was separated with 3 μl of bromophenol loading dye by 8% non-denaturing polyacrylamide gel electrophoresis (PAGE) against 5 μl λHindIII size-fragmented DNA marker (Promega, Southampton, UK).

### MVR-PCR primers (Oswel Synthesis, Southampton, UK)

31A 5′ CCCTTTGCACGCTGGACGGGTGGCG 3′
TAG II 5′ GACACTCACAAAGAACAACGGACA 3′
31-TAG II AC
5′ GACACTCACAAAGAACAACGGACATCTGTGGGAG-GTGGAC 3′
31-TAG II GT
5′ GACACTCACAAAGAACAACGGACATCTGTGGGAG-GTGGGT 3′

### MVR-PCR amplification

Each 25 μl PCR reaction used 100 ng genomic DNA, 1.5 mM magnesium chloride ($MgCl_2$, Biogene), 2% DMSO, 0.2 mM each of dATP, dCTP, dGTP and dTTP, 1 μM 31A, 1 μM TAG II, and 20 nM 31-TAG II AC or GT, and 0.5 U Bio/Polythermase™ according to the manufacturers conditions. Products were amplified in a Techne PCR thermocycler with one denaturing step of 94˚C for 3 minutes, 52˚C for 30 seconds and 72˚C for 15 seconds, 10 cycles of 94˚C for 30 seconds, 52˚C for 30 seconds and 72˚C for 15 seconds, 25 cycles of 94˚C for 30 seconds, 62˚C for 30 seconds and 72˚C for 15 seconds, finally, extension at 72˚C for 2 minutes. The products of PCR were separated immediately by electrophoresis on a 8% non-denaturing polyacrylamide gel with a PhiX174 Hinf I DNA marker (Promega, Southampton, UK). The gel was then stained with ethidium bromide (Sigma) and photographed under ultraviolet excitation.

### Microsatellite analysis

Replication error analysis was performed using PCR amplification of 10 microsatellites. These were the dinucleotide repeats D2S123, D5S404, D7S519, D8S255, D10S197, D11S904, D13S175, D15S120, D17S787, and D18S58 (Lothe et al, 1993; Thibodeau et al, 1993; Oswel Synthesis). The PCR conditions were as

**Table 1** Conditions for microsatellite analysis

| PCR | Primer amount (ng) | [$MgCl_2$] (mM) | [DMSO] (%) | Units bio/polythermase | Amount target (μl) | Final volume (μl) | Annealing temp (˚C) | Expected product size (bp) |
|---|---|---|---|---|---|---|---|---|
| D2S123 | 75 | 2.5 | 1 | 0.1 | 0.5 | 25 | 60 | 197–227 |
| D5S404 | 75 | 2.75 | 0 | 0.1 | 0.5 | 25 | 60 | 180–198 |
| D7S519 | 75 | 2 | 0 | 0.1 | 0.5 | 25 | 56 | 256–268 |
| D8S255 | 75 | 2.75 | 0 | 0.1 | 0.5 | 25 | 56 | 107–129 |
| D10S197 | 75 | 2 | 0 | 0.1 | 0.5 | 25 | 65 | 161–173 |
| D11S904 | 75 | 2 | 0 | 0.1 | 0.5 | 25 | 58 | 185–201 |
| D13S175 | 75 | 2 | 0 | 0.1 | 0.5 | 25 | 60 | 101–113 |
| D15S120 | 75 | 2 | 0 | 0.1 | 0.5 | 25 | 60 | 121–145 |
| D17S787 | 75 | 2.75 | 0 | 0.1 | 0.5 | 25 | 65 | 138–166 |
| D18S58 | 75 | 2.5 | 2 | 0.1 | 0.5 | 25 | 53 | 144–160 |

© 2001 Cancer Research Campaign described in Table 1, with 0.2 mM dNTPs and 1 × PCR buffer (Biogene). The tubes were placed in an automatic thermal cycler and denatured for 3 minutes at 94˚C followed by 35 repeated cycles of 94˚C for 30 seconds, annealing at the temperature indicated in Table 1 for 15 seconds and extension at 72˚C for 15 seconds. This was followed by a final elongation step at 72˚C for 2 minutes. A negative PCR reaction control using sterile water in place of genomic DNA was used in all cases. 10 μl of each product was separated by 8% PAGE with 3 μl bromophenol loading dye and visualised by staining with ethidium bromide.

### Heteroduplex analysis and MMR gene sequencing

Genomic DNA (100 ng) was amplified using the relevant primers for the 19 exons of hMLH1 and the 16 exons of hMSH2 using the conditions described (Beck et al, 1997). The PCR products were heated to 95˚C for 3 min then cooled to 37˚C over a period of 30 min to form heteroduplexes before running 10 μl of sample on a 24 cm Hydrolink gel (AT Biochem, UK). Heteroduplex bands were visualised by ethidium bromide staining.

To obtain the sequence, PCR of the relevant region was performed using a biotin tagged forward primer (Oswel Synthesis). A total of 100 μl of the PCR product was run on a 1% NuSieve (Flowgen, Lichfield, UK) low melting-point agarose gel, the PCR band was excised from the gel, and the biotinylated template strand for sequencing was isolated using Dynabeads (Dynal, Liverpool, UK). Sequencing was carried out using a Sequenase Version 2.0 T7 DNA Polymerase kit (United States Biochemical, Cleveland, Ohio, USA), labelling with $^{35}$S-αCTP. Sequencing products were separated on a 6% denaturing urea/polyacrylamide gel and visualised by X-ray autoradiography or on a Fuji phosphoimager.

### RESULTS

Tumour and normal mucosa was collected from 33 cases of colorectal neoplasia. Of these, 27 were sporadic and 6 from suspected HNPCC patients (most either MMR gene mutation or Amsterdam criteria positive family history). All patients apart from 2 of the sporadic group had histology reports confirming adenocarcinoma; numbers 17 and 21 in the sporadic group had adenomata with dysplasia (Table 2). Analysis of 10 microsatellite markers in the 27 sporadic tumours revealed 3 (11%) to demonstrate MSI+ in greater than 40% of loci (MSI-high) (Table 2). Loss of heterozygosity (LOH) was also observed with some markers (Table 2). An additional one tumour (3%) demonstrated MSI in less than 40% of markers (MSI-low).

After separation of the products of MVR-PCR on a gel by electrophoresis and visualisation, complementary ladders are revealed from each of the two repeat unit specific primers, for normal and tumour DNA. Therefore 4 lanes for each patient are seen on the gel (Figure 1). MVR-PCR for the 3 MSI-high sporadic tumours demonstrated consistent alterations compared with normal DNA on several occasions. These alterations included smearing and band loss of the products from tumour DNA. LOH at the D7S21 locus in the tumours can be ruled out as in some positions in the gel both AC or GT bands co-exist. The co-migration of AC and GT bands is only possible if 2 distinct products of the same size are produced from 2 loci. With the 23 MSI-negative tumours and the one MSI-low tumour the above marked changes were not seen

**Table 2** Results for sporadic colorectal cancers

| Patient number | Age | Sex | Site | Dukes stage | Tumour differentiation | MSI (altered loci, see legend) | LOH (loci) | MVR-PCR |
|---|---|---|---|---|---|---|---|---|
| 1 | 79 | M | Sig | C | Poor | – | | Neg |
| 2 | 83 | M | Rec | C | Mod | – | 11 | Neg |
| 3 | 80 | F | Rec | B | Mod | – | | Neg |
| 4 | 77 | F | Rec | B | Well | – | | Neg |
| 5 | 76 | F | Rec | B | Mod | – | | Neg |
| 6 | 75 | M | Left | B | Poor | – | | Neg |
| 7 | 67 | F | RS | A | Mod | – | 11 | Neg |
| 8 | 79 | M | Right | D | Poor | – | | Neg |
| 9 | 72 | M | Rec | B | Mod | – | 5 | Neg |
| 10 | 84 | F | Tr | C | Mod | 2,5,8,10,11,13,1,5,17,18 | | Pos |
| 11 | 69 | F | Right | B | Mod | – | | Neg |
| 12 | 75 | M | Left | C | Poor | – | 5,8,17 | Neg |
| 13 | 80 | F | Right | B | Well | – | | Neg |
| 14 | 67 | M | Sig | B | Poor | – | | Neg |
| 15 | 74 | M | Rec | C | Mod | – | | Neg |
| 16 | 68 | F | Rec | C | Mod | – | | Neg |
| 17 | 50 | F | Rec | Polyp | Dyspl TA | – | | Neg |
| 18 | 75 | M | RS | D | Mod | – | | Neg |
| 19 | 69 | M | Tr | B | Well | – | 5 | Neg |
| 20 | 77 | M | Sig | C | Mod | – | 8,17 | Neg |
| 21 | 57 | M | Sig | Polyp | Dyspl Ad | – | | Neg |
| 22 | 58 | F | Rec | D | Poor | – | | Neg |
| 23 | 65 | M | Rec | C | Mod | – | 8 | Neg |
| 24 | 67 | M | Rec | C | Poor | 8,15 | | Neg |
| 25 | 78 | F | Tr | B | Mod | – | | Neg |
| 26 | 63 | F | Right | C | Mod | 7,15,17,18 | 2,13 | Pos |
| 27 | 77 | M | Sig | D | Poor | 10,13,15,18 | 2,8 | Pos |

Site (of tumour): Sig. = sigmoid, Rec. = rectum, R.S. = recto-sigmoid, Tr. = transverse. Grade: Poor = poorly differentiated, Mod. = moderately differentiated, well = well differentiated, Dyspl. T.A., = dysplastic tubulovillous adenoma, Dyspl. Ad. = dysplastic adenoma. MSI / LOH LOCI: 2 = D2S123, 5 = D5S404, 7 = D7S519, 8 = D8S255, 10 = D10S197, 11 = D11S904, 13 = D13S175, 15 = D15S120, 17 = D17S787, 18 = D18S58.

*© 2001 Cancer Research Campaign*

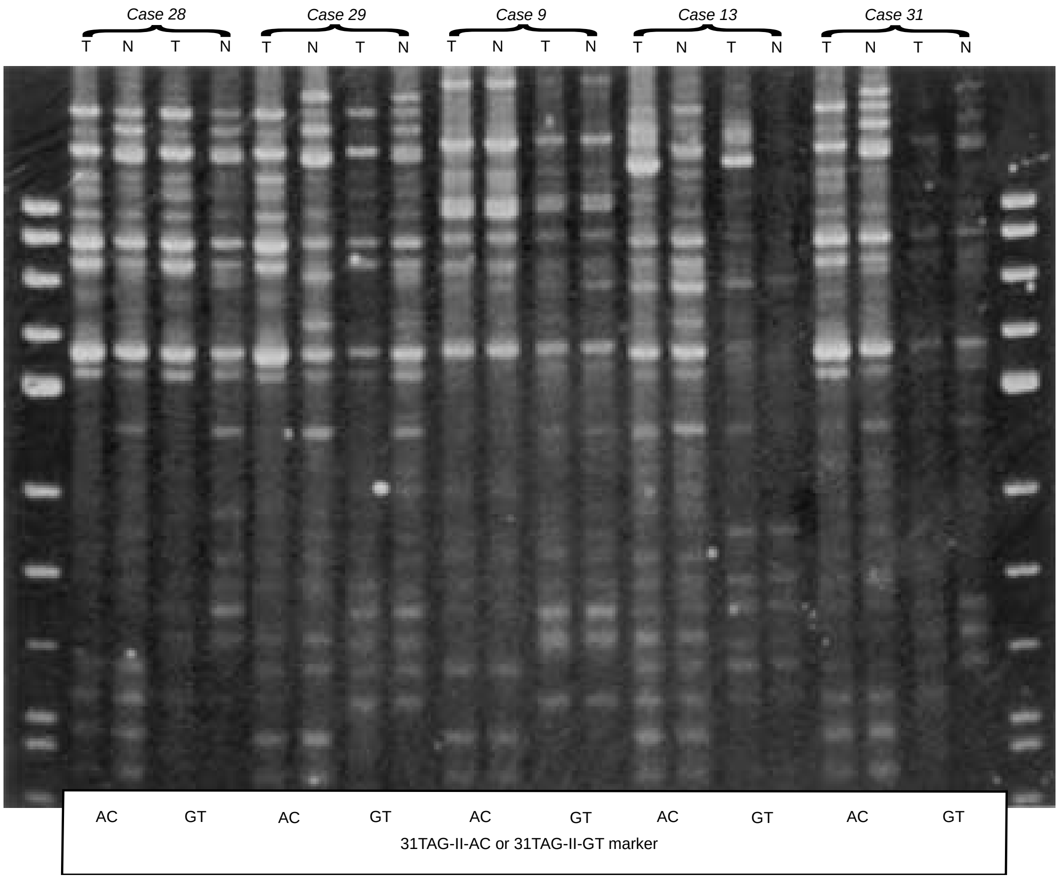

**Figure 1** DNA from fresh snap-frozen tumour (T) / normal (N) tissues of cases 9 and 13 (MSI stable), and cases 28, 29 and 31 of the HNPCC group (MSI high by microsatellite analysis). For cases 9 and 13 no differences were observed between the tumour and normal bands for either reaction (31 TAG-II-AC or -GT). For cases 28, 29 and 31, both smearing and band loss is seen in the tumour DNA compared to the normal DNA. The marker (lanes 1 and 22) is PhiX174 Hinf I

between the products from tumour and normal DNA (Figure 1) although an occasional aberrant band was observed in tumour DNA.

In the 6 cases with suspected HNPCC, 3 were shown by sequencing to have mutations in the MMR gene hMLH1 and 1 in hMSH2. In the remaining 2 a mutation could not be identified in spite of intensive analysis, including hPMS2 using heteroduplex/SSCP analysis. One had a kindred satisfying the Amsterdam criteria and the remaining one had right-sided colorectal cancer at 30 years of age but the family history did not meet the Amsterdam Criteria.

Five of the 6 suspected HNPCC cases yielded positive results from analysis by MVR-PCR (Figure 1 and Table 3) with band loss and smearing of product from tumour DNA. Only 2 of the 6 demonstrated MSI in 4 out of 10 loci (MSI-high) and an additional one in 3

**Table 3** Results of MVR-PCR and microsatellite analysis on patients with suspected HNPCC. All except patient 33 demonstrated features suggesting instability at minisatellite loci. Two of the 3 cases with MMR gene mutations were MSI stable (neg). Of the 2 Amsterdam positive (MMR negative) cases, 1 was MSI high. For MSI loci see Table 2

| Patient number | Age | Sex | Site | Dukes stage | Amsterdam criteria | MSI (loci altered) | MMR gene mutations | MVR-PCR |
|---|---|---|---|---|---|---|---|---|
| 28 | 30 | M | R colon | B Later liver metastases | Neg | 2,7,11,15 | None detected | Pos |
| 29 | 63 | F | R colon | C | Pos | 7,13,15 | HMLH1 exon 19 stop mutation G > A @base 2135 | Pos |
| 30 | 39 | F | R colon | A | Pos | Neg | HMLH1 exon 1 2 bp insertion +AA @ base 105 | Pos |
| 31 | 51 | M | R colon | B | Pos | 2,10,11,13,15,18 | HMSH2 exon 15 7 bp deletion CTAATTTCCC to CCC @codon 836 | Pos |
| 32 | 28 | M | R colon | B | Neg | Neg | HMLH1 exon 16 3 bp deletion GAAGAAGAAG to GAAGAAG @codon 616/617/618 | Pos |
| 33 | 51 | F | Sigmoid colon | N/K | Pos | Neg | None detected | Neg |

© 2001 Cancer Research Campaign out of 10 (MSI-low). Of the 3 that were MSI stable, 2 had mutations in hMLH1 (Table 3). The one patient in this group who was stable on MVR-PCR was MSI stable and no mutation could be detected.

In summary, therefore, 8 out of the 33 patients had mismatch repair defects as demonstrated by either MSI or MMR mutation. All 8 of these patients demonstrated minisatellite instability using MVR-PCR.

### DISCUSSION

In this study we have shown that MSI-high sporadic colorectal tumours and tumours from patients with germline hMLH1 mutations or other convincing features of HNPCC invariably manifest minisatellite instability by the application of MVR-PCR. This is easily visualised as smearing and band loss comparing normal and tumour DNA. No MSI negative or MSI-low sporadic tumours exhibited these features. Further, in 2 patients with demonstrated hMLH1 mutations and minisatellite instability, MSI was not found even with the use of 10 microsatellite markers. The only patient suspected of having HNPCC, on the basis of clearly satisfying the Amsterdam criteria, who did not manifest instability on MVR-PCR had a left-sided cancer, was MSI stable and had no detectable mutation in hMLH1, hMSH2 or hPMS2. Possible explanations for this observation include a sporadic colorectal cancer in this patient or insufficient tumour material in the sample examined. These observations raise the possibility that MMR deficiency may be easily and reliably detected by MVR-PCR, a technique that utilises a pair of PCR reactions. The findings of this study also may give additional insight into the nature of DNA instability in tumours with MMR deficiency.

Conventionally the DNA instability observed in colorectal cancer is detected by the PCR amplification of microsatellites which are di-and tri-nucleotide repeats found scattered throughout the human genome and are characteristically found in non-coding DNA sequences (Aaltonen et al, 1993). Since microsatellite alleles are short in sequence length (up to 100 bp), individual loci may not necessarily manifest DNA instability even though the genome as a whole may be affected. Therefore several microsatellite loci are amplified and analysed per case and if a proportion manifest differences between tumour and normal DNA, then microsatellite instability (MSI) is inferred. Until recently (Bocker et al, 1997; Dietmaier et al, 1997; Boland et al, 1998), there has been no consensus as to how many microsatellite loci must be altered to diagnose the MSI phenotype. The likelihood of alteration in microsatellites varies from 55 to 91% in different loci in familial colon cancer (Aaltonen et al, 1993), and between 12 and 28% in sporadic colorectal tumours (Aaltonen et al, 1993; Thibodeau et al, 1993). MMR gene mutations can be found in approximately 70% of patients with familial colorectal cancer (Aaltonen et al, 1994). In sporadic colorectal cancer with MSI (High) MMR gene mutations are not commonly found but there normally is loss of hMLH1 expression due to epigenetic silencing by methylation of CpG in the hMLH1 promoter (Kane et al, 1997).

In both minisatellites and microsatellites allelic variability is a feature of the number of tandem repeats (Aaltonen et al, 1993). Minisatellites have a total array size of 0.5–30 kb and are also widespread in the human genome (Jeffreys et al, 1991). Such loci may be amplified by PCR. The usefulness of the information generated is limited by error prone estimates of allele length and equivocal allele identification (Jeffreys et al, 1991). An alternative method of typing minisatellite loci is to assay the sequence variation of tandem repeat units. Minisatellite alleles vary not only in the repeat copy number but also in the interspersion pattern of variant repeat units along alleles (Jeffreys et al, 1991). In certain rare loci two classes of repeat unit differ only by a single base substitution that either creates or destroys a *Hae*III restriction endonuclease site (Neil and Jeffreys, 1993). MVR-PCR is a further means of analysis of DNA polymorphism in minisatellite alleles by the study of repeat unit sequence variation (Jeffreys et al, 1991). MVR-PCR generates an extraordinarily variable but unambiguous pattern used in a variety of applications (Jeffreys et al, 1991). The MS31A locus conforms to these requirements for maximum informativeness in a MVR locus.

DNA mismatches occur during mitotic replication either by incorrect base pairings or by slippage of DNA polymerase on the template strand. Slippage is most likely to occur during replication of long repeating sequences and results in more or fewer copies of the repeat (Neil and Jeffreys, 1993). The mechanisms for DNA mismatch repair have been extensively characterized in prokaryotes (*E. coli*) (Strand et al, 1993) and homologous repair systems have also been identified in eukaryotes (Prolla et al, 1994). These repair processes have also been found in human cell extracts (Thomas et al, 1991) where they have the ability to repair mismatch loops up to 16 bases (Umar et al, 1994). Loops of this size may develop if the mismatched sequence is longer than the 2 to 3 base pair sequences observed in bacteria.

Prior to the discovery of the RER phenotype and its link with microsatellites, somatic alterations in tumour DNA minisatellites had been recognised (Thein et al, 1987; Armour et al, 1989). Amplification of minisatellite alleles from tumour DNA revealed band losses, intensification and novel bands when compared to constitutional DNA. Interestingly these were found in a small proportion of colorectal carcinomas. These observations were made prior to the discovery of DNA instability and mismatch repair genes and their role in colorectal carcinogenesis. Spontaneous alterations in allele length have previously been observed in colorectal cancer (Hoff-Olsen et al, 1995).

The analysis of microsatellite markers in this study was performed before the publication of the recent consensus (Dietmaier et al, 1997). Although the microsatellites used here have been well evaluated we fully accept that we may have detected MSI in more samples using the consensus panel, particularly if the BAT26 locus had been probed. We (unpublished) and others (Shitoh et al, 1998) have found this locus particularly informative. Nevertheless the principal conclusion of this study would have been unaffected by using more probes as we found minisatellite instability in all of the tumours with a MMR gene mutation and only in MSI (high) sporadic tumours. To date only a limited number of genetic alterations have been examined in the context of MMR deficiency, specifically those associated with di- and tri-nucleotide repeats. Although the mechanisms may not be fully understood it appears that MMR deficiency can influence sequence heterogeneity in longer repeat sequences, such as those detected by MVR-PCR. In this study using MS31A, the repeats were still relatively short (20 bp). Further work is required to determine whether the same phenomenon may be observed in other minisatellites, including those with a longer repeat sequence. This will give insight into whether the range of genetic alteration found in MMR deficiency needs to be revised.

In conclusion, this study supports the further evaluation of MVR-PCR, which is a simple and reproducible technique involving 2 PCR reactions, as compared to the 6 required if the

© 2001 Cancer Research Campaign consensus panel is used (Boland et al, 1998). However this study was performed using frozen tissue. We are currently performing work to determine whether the technique may be equally applied to formalin-fixed tissue, essential if it is to have a widespread application.

### REFERENCES

Aaltonen LA, Peltomäki P, Leach FSP, Sistonen P, Pylkkänen L, Mecklin J-P, Järvinen H, Powell SM, Jen J, Hamilton SR, Petersen GM, Kinzler KW, Vogelstein B and de la Chapelle A (1993) Clues to the pathogenesis of familial colorectal cancer. *Science* **260**: 812–815

Aaltonen LA, Peltomäki P, Mecklin J-P, Järvinen H, Jass JR, Green JS, Lynch HT, Watson P, Tallqvist G, Juhola M, Sistonen P, Hamilton SR, Kinzler KW, Vogelstein B and de la Chapelle A (1994) Replication errors in benign and malignant tumors from hereditary nonpolyposis colorectal cancer patients. *Cancer Res* **54**: 1645–1648

Armour JAL, Patel I, Thein SL, Fey MF and Jeffreys AJ (1989) Analysis of somatic mutations at human minisatellite loci in tumours and cell lines. *Genomics* **4**: 328–334

Beck NE, Homfray TFR, Frayling IM, Hodgson SV, Harocopos C and Bodmer WF (1997) Use of SSCP analysis to identify germline mutations in HNPCC families fulfilling the Amsterdam criteria. *Human Genetics* **99**: 219–224

Bocker T, Diermann J, Friedl W, Gebert J, Holinski-Feder E, Karner-Hanusch J, von Knebel-Doeberitz M, Moeslein G, Schackert H-K, Wirtz H-C, Fishel R and Rüschoff J (1997) Microsatellite instability analysis: multicenter study for reliability and quality control. *Cancer Research* **57**: 4739–4743

Boland CR, Thibodeau SN, Hamilton SR, Sidransky D, Eshleman JR, Burt RW, Meltzer SJ, Rodrigez-Bigas MA, Fodde R, Ranzani GN and Srivastava SA (1998) National Cancer Institute Workshop on Microsatellite Instability for Cancer Detection and Familial Predisposition: Development of International Criteria for the Determination of Microsatellite Instability in Colorectal Cancer. *Cancer Rese* **58**: 5248–5257

Dietmaier W, Wallinger S, Bocker T, Kullman F, Fishel R and Rüschoff J (1997) Diagnostic Microsatellite instability: Definition and correlation with mismatch repair protein expression. *Cancer Research* **57**: 4749–4756

Fearon ER and Vogelstein B (1990) A genetic model for colorectal tumorigenesis. *Cell* **61**: 759–767

Fishel R, Lescoe MK, Rao MRS, Copeland NG, Jenkins NA, Garber J, Kane M and Kolodner R (1993) The human mutator gene homolog MSH2 and its association with hereditary nonpolyposis colon cancer. *Cell* **75**: 1027–1038

Hoff-Olsen P, Meling GI and Olaisen B (1995) Somatic mutations in VNTR-locus in human colorectal carcinomas are associated with microsatellite instability. *Human Mutation* **5**: 329–332

Ionov Y, Peinado MA, Malkhosyan S, Shibata D and Perucho M (1993) Ubiquitous somatic mutations in simple repeated sequences reveal a new mechanism for colonic carcinogenesis. *Nature* **363**: 558–561

Jeffreys AJ, MacLeod A, Tamaki K, Neil DL and Monckton DG (1991) Minisatellite repeat coding as a digital approach to DNA typing. *Nature* **354**: 204–209

Kane MF, Loda M, Gaida GM, Lipman J, Mishra R, Goldman H, Jessup JM and Kolodner R (1997) Methylation of the hMLH1 promoter correlates with lack of expression of hMLH1 in sporadic colon tumors and mismatch repair-defective human tumor cell lines. *Cancer Res* **57**: 808–811

Lothe RA, Peltomäki P, Meling GI, Aaltonen LA, Nyström-Lahti M, Pylkkänen L, Heimdal K, Andersen TI, Møller P, Rognum TO, Fosså SD, Haldorsen T, Langmark F, Brøgger A, de la Chapelle A and Børresen A-L (1993) Genomic instability in colorectal cancer: relationship to clinicopathological varibles and family history. *Cancer Res* **53**: 5849–5852

Nakamura Y, Leppert M, O'Connell P, Wolff R, Holm T, Culver M, Martin C, Fujimoto E, Hoff M, Kumlin E and White R (1987) Variable number of tandem repeat (VNTR) markers for human gene mapping. *Science* **235**: 1616–1622

Neil DL and Jeffreys AJ (1993) Digital DNA typing at a second hypervariable locus by minisatellite variant repeat mapping. *Human Molecular Genetics* **2**: 1129–1135

Papodopoulos N, Nicolaides NC, Wei Y-F, Ruben SM, Carter KC, Rosen CA, Haseltine WA, Fleischmann RD, Fraser CM, Adams MD, Venter JC, Hamilton SR, Petersen GM, Watson P, Lynch HT, Peltomäki P, Mecklin J-P, de la Chapelle A, Kinzler KW and Vogelstein B (1994) Mutation of a mutL homolog in hereditary colon cancer. *Science* **263**: 1625–1629

Prolla TA, Christie D-M and Liskay RM (1994) Dual requirement in yeast DNA mismatch repair for MLH1 and PMS2, two homologs of the bacterial mutL gene. *Molecular and Cell Biology* **14**: 407–415

Shitoh K, Konishi F, Masubuchi S, Senba S, Tsukamoto T and Kanazawa K (1998) Important microsatellite markers in the investigation of replication errors (RER) in colorectal carcinomas. *Japanese Journal of Clinical Oncology* **28**: 538–541

Strand M, Prolla TA, Liskay RM and Petes TD (1993) Destabilisation of tracts of simple repetitive DNA in yeast by mutations affecting DNA mismatch repair. *Nature* **365**: 274–276

Thein SL, Jeffreys AJ, Gooi HC, Cotter F, Flint J, O'Conner NTJ, Weatherall DJ and Wainscoat JS (1987) Detection of somatic changes in human cancer DNA by DNA fingerprint analysis. *British Journal of Cancer* **55**: 353–356

Thibodeau SN, Bren G and Schaid D (1993) Microsatellite instability in cancer of the proximal colon. *Science* **260**: 816–819

Thomas DC, Roberts JD and Kunkel TA (1991) Heteroduplex repair in extracts of human Hela cells. *Journal of Biological Chemistry* **266**: 3744–3751

Umar A, Boyer JC and Kunkel TA (1994) DNA loop repair by human cell extracts. *Science* **266**: 814–816

*© 2001 Cancer Research Campaign*
